# High tide or low tide: the transport and metabolism of mitochondrial nucleotides

**DOI:** 10.1042/BCJ20253237

**Published:** 2025-08-18

**Authors:** Thomas MacVicar

**Affiliations:** 1 Cancer Research UK Scotland Institute, Glasgow, United Kingdom; 2 School of Cancer Sciences, University of Glasgow, Glasgow, United Kingdom

**Keywords:** nucleotides, metabolism, mitochondria, nucleotide transport, nucleotide salvage, mitochondrial disease

## Abstract

Mitochondria are multifaceted organelles that support numerous cellular metabolic pathways, including the biosynthesis of nucleotides required for cell growth and proliferation. Owing to an ancient endosymbiotic origin, mitochondria contain multiple copies of their own genome and therefore demand sufficient (deoxy)nucleotides in the mitochondrial matrix for DNA replication and transcription into RNA. Disturbed mitochondrial deoxynucleotide homeostasis can lead to a decline in mitochondrial DNA abundance and integrity, causing mitochondrial diseases with diverse and severe symptoms. Mitochondrial nucleotides are not only required for nucleic acid synthesis but also for bioenergetics and mitochondrial enzymatic activity. This review first explores how mitochondria supply energy and anabolic precursors for nucleotide synthesis and how the mitochondrial network influences the spatial control of cellular nucleotide metabolism. Then follows an in-depth discussion of the mechanisms that supply mitochondria with sufficient and balanced nucleotides and why these mechanisms are relevant to human mitochondrial disease. Lastly, the review highlights the emergence of regulated mitochondrial nucleotide supply in physiological processes including innate immunity and discusses the implications of dysregulated mitochondrial and cytosolic nucleotide homeostasis in pathophysiology.

## Introduction

Nucleotides are the building blocks of all genetic material and are key substrates or cofactors in cellular metabolism and signalling. The nucleotides we recognise today may have evolved from pre-nucleotides that arose during prebiotic geochemical evolution [1]. They consist of three modules: a phosphate group, ribose sugar and pyrimidine or purine heterocyclic aromatic nucleobase, which are bound together by condensation. Nucleosides do not contain a phosphate group, whereas nucleotides are mono-, di- or tri-phosphorylated at the 5′ hydroxyl group of the ribose sugar. The pyrimidine nucleobases cytosine, uracil and thymine have a pyrimidine ring, while the purine nucleobases adenine and guanine have a fused pyrimidine–imidazole group. Ribonucleoside triphosphates (NTPs) or deoxyribonucleoside triphosphates (dNTPs) differ solely by the presence or absence of a hydroxyl group at the 2′ carbon of ribose sugar. NTPs and dNTPs polymerise to form RNA and DNA, respectively.

Nucleotides are synthesised *de novo* from various sources of carbon and nitrogen or scavenged from precursor nucleobases and nucleosides in purine and pyrimidine nucleotide salvage pathways [2]. Purine nucleotide salvage starts with phosphoribosyl transfer onto nucleobases (hypoxanthine, adenine and guanosine) to generate inosine, adenosine and guanosine monophosphates. Conversely, pyrimidine nucleotide salvage starts with the phosphorylation of nucleosides (uridine, cytidine and thymidine) into their respective nucleoside monophosphates because mammalian cells cannot utilise nucleobases for pyrimidine nucleotide salvage [3,4]. Crucially, nucleotide homeostasis is also maintained by nucleotide degradation pathways [5,6]. These anabolic and catabolic routes provide careful regulation of nucleotide levels and are influenced by the metabolic and developmental status of cells. Classically, proliferating cells are described to up-regulate *de novo* synthesis of nucleotides to meet demands for nuclear DNA replication. Increased dNTP levels are achieved largely by enhanced activity of ribonucleotide reductase (RNR), which converts ribonucleoside diphosphates (NDPs) into deoxyribonucleoside diphosphates (dNDPs) [7]. Conversely, differentiated and post-mitotic cells recycle pyrimidine and purine nucleobases and nucleosides in the less energetically costly salvage pathways. However, the balance between *de novo* synthesis and salvage is not clear cut and can be rewired in healthy or diseased tissues. For instance, proliferating tumours often depend on nucleotide salvage, particularly in conditions that may limit *de novo* pathway activity including nutrient deprivation and exposure to chemotherapy [8,9]. Aberrant nucleotide metabolism causes numerous metabolic and autoimmune diseases, and the pharmacological inhibition of nucleotide metabolism remains a common approach in the treatment of pathologies, including cancer. Several recent reviews provide excellent overviews of cellular nucleotide metabolism in health and disease [2,6,10].

This review focuses on the multilayered relationship between nucleotides and the essential metabolic organelles called mitochondria. Mitochondria provide cells with energy and metabolites to produce nucleotides, while they themselves demand a constant supply of nucleotides for the maintenance and expression of mitochondrial genes and for enzymatic activity. The following sections highlight why mitochondria are core hubs in nucleotide biosynthetic pathways and explore the disease-relevant mechanisms that govern mitochondrial nucleotide supply.

## Mitochondria provide resources and platforms for constructing nucleotides

**Figure 1:**
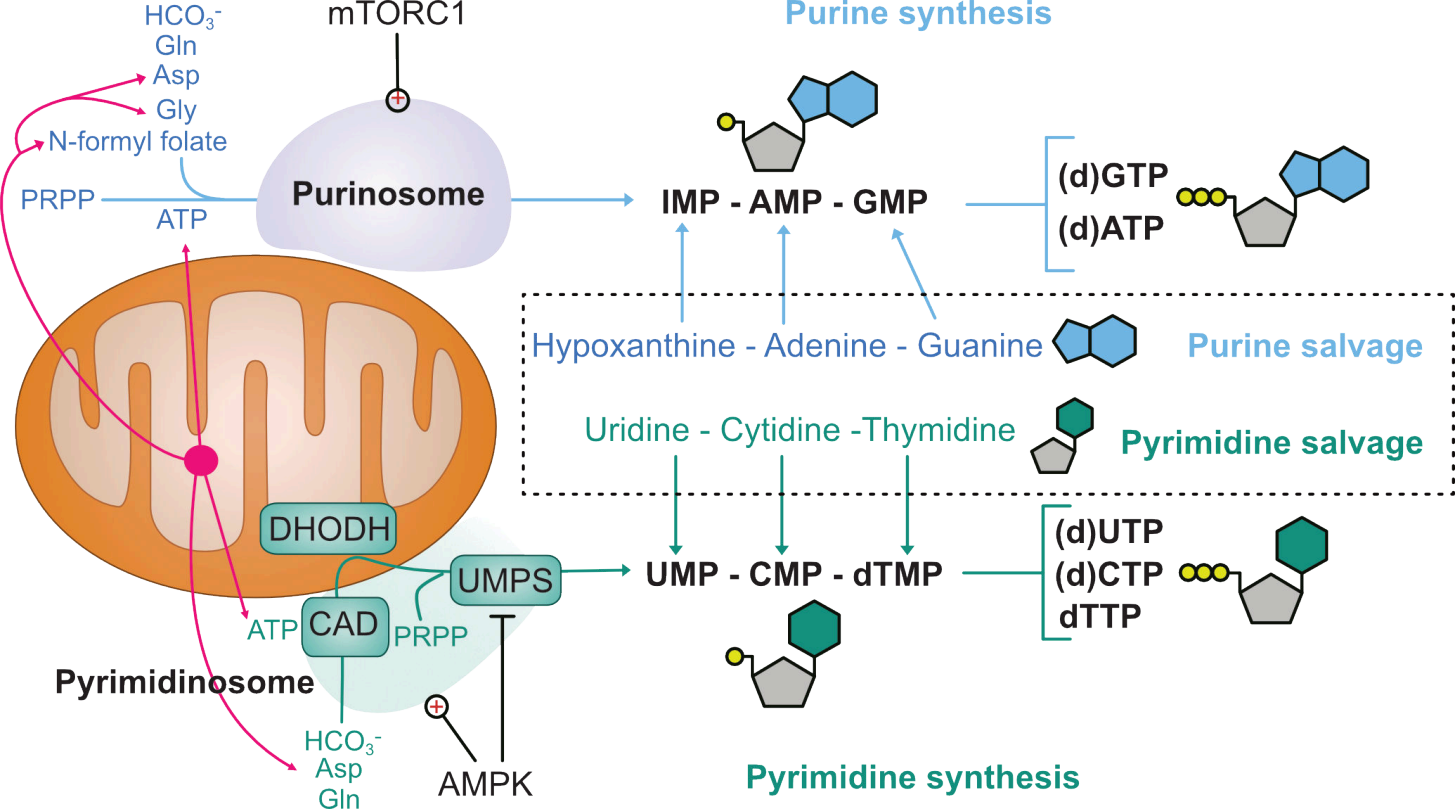
Mitochondria and cytosolic nucleotide metabolism. ATP and precursor metabolites are exported from mitochondria for *de novo* synthesis of nucleotides (pink arrows). Mitochondria are also platforms for the purinosome and pyrimidinosome. The purinosome contains six enzymes in the *de novo* purine biosynthesis pathway. Purinosome interaction with mitochondria is positively regulated by mTORC1. The pyrimdinosome contains the multi-subunit enzyme CAD and UMPS, which localise to mitochondria in an AMPK-dependent manner. AMPK activation increases flux through the pyrimidine biosynthesis pathway up to the production of orotate because AMPK also inhibits UMPS. Cytosolic pools of (d)NTPs are also maintained by nucleotide salvage from purine and pyrimidine nucleosides. Simplified nucleotide structures are depicted with phosphate (yellow), ribose (grey) and nucleobase (blue/green) groups. AMPK, AMP-activated protein kinase; Asp, aspartate; CAD, carbamoyl-phosphate synthetase 2, aspartate transcarbamoylase, and dihydroorotase; DHODH, dihydroorotate dehydrogenase; Gln, glutamine; Gly, glycine; PRPP, phosphoribosyl pyrophosphate; mTORC1, mammalian target of rapamycin complex 1; UMPS, uridine monophosphate synthase.

Mitochondria are double-membraned organelles that play diverse roles in metabolic and signalling pathways besides the production of ATP via oxidative phosphorylation [11]. For instance, they are essential for catabolic and anabolic metabolism, redox homeostasis, calcium and ion homeostasis, iron–sulphur cluster biogenesis and cell death signalling. The *de novo* synthesis of purines and pyrimidines depends on ATP and biosynthetic intermediates generated by mitochondria [5]. Furthermore, recent evidence suggests that cytosolic nucleotide synthesis enzyme complexes localise to mitochondria in a regulated manner, which argues that mitochondria support the spatial regulation of nucleotide biosynthesis (Figure 1).

 Purine biosynthesis requires ATP, amino acids and one-carbon units as sources of carbon and nitrogen, many of which come from mitochondria [6]. For instance, the mitochondrial tetrahydrofolate cycle produces glycine and formyl units for purine biosynthesis [12]. The mitochondrial tetrahydrofolate cycle is enhanced by mammalian target of rapamycin complex 1 (mTORC1), which stimulates anabolic metabolism to meet increased demands for nucleotide synthesis [12,13]. Stimulation of mTORC1 in cultured cells also drives the assembly and mitochondrial association of cytosolic purine synthesis enzymes [14]. Mitochondrial association of this multi-enzyme metabolon, named the purinosome, is supported by microtubule trafficking of purine biosynthesis enzymes [15] and is increased during hypoxia [16]. Mitochondria–purinosome colocalisation may channel mitochondrial metabolites, including glycine and aspartate, into the purine biosynthesis pathway [17,18].

 Pyrimidine biosynthesis is directly coupled to mitochondrial respiration and bioenergetics via dihydroorotate dehydrogenase (DHODH) [19]. DHODH localises to the intermembrane space (IMS) where it is bound to the inner mitochondrial membrane (IMM). DHODH oxidises dihydroorotate to orotate, which is the only *de novo* nucleotide synthesis reaction to occur within mitochondria [20]. Inhibition of DHODH depletes pyrimidines, which may prove to be a viable strategy to target certain cancers, especially if combined with immunotherapy [21]. DHODH catalytic activity reduces ubiquinone (also known as coenzyme Q_10_) to ubiquinol, which supplies electrons to complex III in the electron transport chain. Consequently, inhibition of electron transport and depletion of ubiquinone electron acceptors block pyrimidine biosynthesis [22]. The electron transport chain is also required for the biosynthesis of aspartate, which is a substrate for pyrimidine and purine biosynthesis [23,24]. Thus, mitochondrial bioenergetic activity is closely linked to nucleotide metabolism.

Recent data demonstrate that pyrimidine biosynthesis enzymes can also colocalise at the outer mitochondrial membrane (OMM) in a multi-enzyme complex termed the pyrimidinosome [25] (Figure 1). Pyrimidinosome formation at a fraction of mitochondria appears to promote substrate channelling of newly synthesised aspartate to pyrimidine production. Like the purinosome, pyrimidinosome interaction with mitochondria is dynamically regulated, which is mediated in this case by the master sensor of energetic stress, AMPK. AMPK is activated by an elevated AMP:ATP ratio and promotes the accumulation of mitochondrial pyrimidinosomes while simultaneously inhibiting the final pyrimidinosome enzyme, uridine monophosphate (UMP) synthase. Increased flux through DHODH and production of orotate may support ubiquinol production in the IMM and allow removal of toxic intermediates of the pyrimidine biosynthesis pathway without increasing UMP synthesis [25]. Further work is necessary to understand the importance of purinosome and pyrimidinosome physical interaction with mitochondria, particularly under metabolic stress. It remains unclear whether the formation and control of these large multi-enzyme metabolons is co-regulated to support balanced purine and pyrimidine production and whether they help funnel newly synthesised nucleotides into mitochondria.

Mitochondria are integrated in the *de novo* nucleotide synthesis pathways, but we understand comparatively little of how mitochondria influence cytosolic nucleotide salvage pathways. Nevertheless, mitochondrial activity can determine the extent to which cells depend on nucleotide salvage. As mentioned above, chronic inhibition of mitochondrial respiration in cultured cells renders cells auxotrophic for the pyrimidine salvage precursor uridine [22,26]. Links between mitochondrial activity and the purine salvage pathway have also been established. For instance, accumulation of fumarate upon mutation of the citric acid cycle enzyme fumarate hydratase inhibits *de novo* purine synthesis and forces kidney cancer cell lines to salvage purines [27]. Genetic and pharmacological inhibition of the electron transport chain also forces cancer cells to depend on purine salvage, which indicates that it may be therapeutically beneficial to target nucleotide salvage in tumours with reduced mitochondrial respiration [28].

## Mitochondria demand a constant supply of nucleotides

The work discussed above highlights that mitochondria are central players in the energetic, anabolic and spatial regulation of cellular purine and pyrimidine synthesis, which is vital for genomic replication and repair, cell metabolism and signalling. We now turn our attention to the main topic of this review and explore how and why mitochondria obtain their own nucleotides and the consequences of disturbed mitochondrial nucleotide supply and metabolism in pathophysiology.

Much of what we understand about mitochondrial nucleotide supply revolves around the small mitochondrial genome and the rare genetic diseases that result from dysfunctional nucleotide supply to mitochondria. Multiple and variable copies of circular mitochondrial DNA (mtDNA) exist in each cell that are packaged into nucleoids spaced throughout the mitochondrial network [29]. The human mitochondrial genome encodes 13 subunits of the electron transport chain, 2 ribosomal RNAs and 22 tRNAs [30]. The nuclear-encoded mtDNA polymerase (POLγ) co-operates with the helicase TWINKLE and mitochondrial single-strand binding protein to replicate mtDNA independent of cell cycle progression [29]. Mitochondria, therefore, require a consistent supply of dNTPs throughout the cell cycle. Furthermore, while there is limited capacity for mtDNA repair in comparison with nuclear DNA, mitochondria do contain a base excision repair pathway that utilises new dNTPs to replace 8-oxoguanine or abasic sites and repair single-stranded DNA breaks [31].

Transcription of mtDNA depends on a supply of NTPs and is catalysed by the nuclear encoded RNA polymerase POLRMT, which generates polycistronic mitochondrial RNA (mtRNA) transcripts that are subsequently processed into messenger RNA, ribosomal RNA and transfer RNA [32]. RNA cannot be imported to the mitochondrial matrix; therefore, mitochondrial NTPs are required to synthesise all rRNA required for the biogenesis and function of mitochondrial ribosomes.

Mitochondria demand NTPs for numerous functions besides polymerisation into RNA. Oxidative phosphorylation requires the import of ADP into the mitochondrial matrix for the electron transfer-coupled synthesis of ATP, which is then exported from mitochondria via the same membrane carrier [33]. In the TCA cycle, the conversion of succinyl coenzyme A to succinate by succinyl coenzyme A synthetase is coupled to substrate-level phosphorylation of ADP and GDP in the mitochondrial matrix [34]. ATP and GTP are required by mitochondrial gene expression enzymes, such as conserved GTPases involved in the assembly of the mitochondrial ribosome [35], and the hydrolysis of mitochondrial GTP is also coupled to other metabolic pathways including the production of phosphophenolpyruvate [36]. Finally, CTP is utilised in the biosynthesis of the mitochondrial-enriched phospholipid cardiolipin in the IMM [37]. In summary, the replication, maintenance and expression of the mitochondrial genome and metabolic activity of mitochondria require a constant supply of nucleotides to the mitochondrial matrix.

## The origins of mitochondrial nucleotides

**Figure 2:**
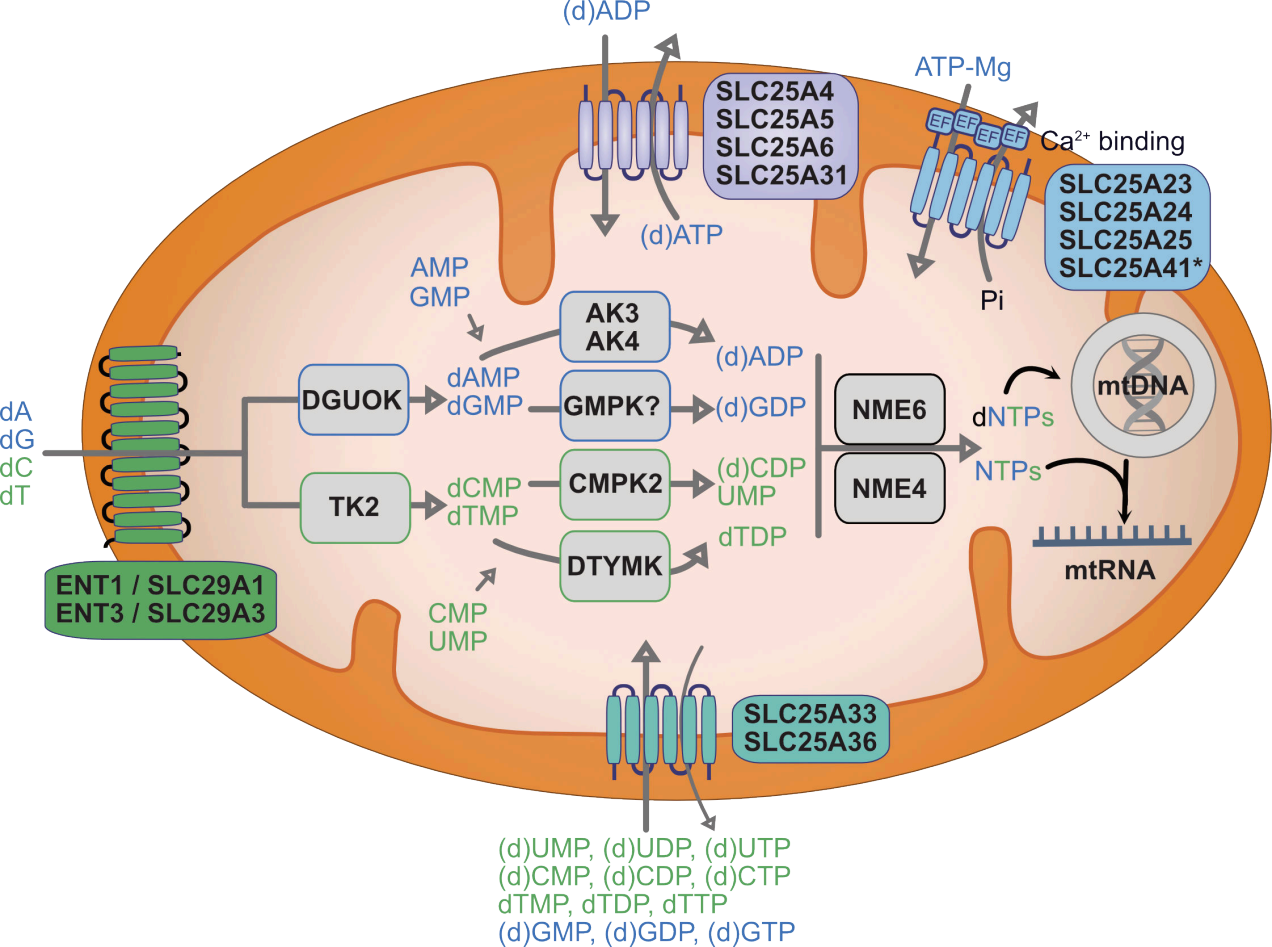
Mitochondrial nucleotide supply routes Mitochondria employ dedicated solute carriers to import deoxynucleosides and (deoxy)nucleotides across the inner mitochondrial membrane. Imported dNTPs and NTPs can be used directly for mtDNA and mtRNA synthesis, respectively. Imported (d)NMPs and (d)NDPs must first be phosphorylated by nucleotide kinases in the mitochondrial nucleotide salvage pathway. DGUOK and TK2 phosphorylate deoxynucleosides specifically. AK3/4, adenylate kinase 3/4; CMPK2, cytidine monophosphate kinase 2; DGUOK, deoxyguanosine kinase; (d)NDP, (deoxy)nucleoside diphosphate; (d)NMP, (deoxy)nucleoside monophosphate; (d)NTP (deoxy)nucleoside triphosphate; dNs, deoxynucleosides; DTYMK, deoxythymidylate kinase; ENT1/3, equilibrative nucleoside transporter 1/3; GMPK, putative guanylate kinase; mtDNA, mitochondrial DNA; mtRNA, mitochondrial RNA; NME4/6, nucleoside diphosphate kinase 4/6; TK2, thymidine kinase 2; *, SLC25A41 does not contain a functional Ca^2+^ binding regulatory domain [52].

The mitochondrial matrix nucleotide pool is distinct from the rest of the cell (Box 1). However, mitochondria lack the enzymes required to synthesise nucleotides *de novo* from carbon and nitrogen sources. Consequently, the ribose and nucleobase groups of all mitochondrial nucleotides are synthesised and fused in the cytosol prior to import into the mitochondrial matrix. There is some evidence of a mitochondrial thymidylate synthase, which is required for the biosynthesis of thymidine monophosphate (dTMP) from deoxyuridine monophosphate (dUMP) and limits the misincorporation of uracil in mtDNA [48,49], but this may be most predominant in plant cell mitochondria [50], and its activity in mammalian mitochondria remains unclear [48,51]. Mitochondria do contain a repertoire of nucleotide salvage kinases in a nucleotide salvage pathway that can synthesise dNTPs starting from deoxynucleosides (dNs) or NTPs starting from NMPs (Figure 2). The importance of sufficient nucleotide supply to mitochondria is reflected by the fact that mutations in several genes associated with nucleotide metabolism cause severe mitochondrial disease. Pathogenic mutation of mitochondrial nucleotide transporters, salvage pathway enzymes and cytosolic enzymes causes mitochondrial dNTP depletion and mtDNA mutation and depletion with diverse tissue-specific outcomes [53]. The following sections explore the mechanisms by which mitochondria obtain sufficient nucleotides and examine the severe human diseases associated with dysfunctional mitochondrial nucleotide transport and metabolism.

Box 1 **Compartmentalised nucleotide pools: a balancing act**

Nucleotide concentrations are carefully regulated within compartmentalised cellular pools. Most studies focus specifically on the abundance and balance of dNTP pools in the cytosol and nucleus because altered levels of total or individual dNTPs can be genotoxic [38]. A variety of techniques have been used to quantify absolute and relative concentrations of nucleotides within cell compartments, including mass spectrometry-based methods and enzymatic dNTP incorporation assays [39]. While efforts are underway to consolidate these data, absolute concentrations of nucleotides vary greatly between cell or tissue types, development or cell-cycle stages and metabolite extraction methods [10,39–41]. In general, for example, dNTP and NTP pools are more abundant in proliferating tumour cells compared with quiescent cells, and the NTP pools are orders of magnitude larger than dNTP pools. The accurate quantification of mitochondrial nucleotides is challenging, and different asymmetries have been reported between individual mitochondrial dNTPs. Interestingly, dGTP appears to be consistently higher in rodent tissue mitochondria where the majority of dGTP is bound to protein [42,43]. It remains unclear whether dNTPs destined for incorporation into mtDNA are somehow maintained close to equimolar concentrations to avoid mutagenesis.

Importantly, mitochondrial nucleotide pools are linked with cytosolic pools via dedicated nucleotide transporters. The concentration of some nucleotides, for instance dATP and dTTP, appears to correlate between both compartments, whereas others do not [41]. In gross terms, the mitochondrial nucleotide pool contributes a small fraction to the total pool of cellular nucleotides, although this will vary depending on mitochondrial mass [41]. Therefore, altered cytosolic nucleotide homeostasis and metabolism has a direct impact on mitochondrial nucleotide pools, while the reverse is less likely. However, as highlighted in this review, excessive uptake of nucleotides into the mitochondrial compartment may affect the abundance and balance of nucleotides in the cytosol and nucleus [44,45]. Cellular nucleotide metabolism can also affect mitochondrial behaviour indirectly; for instance, cytosolic UTP regulates the production of key TCA cofactor thiamine pyrophosphate, which couples cytosolic pyrimidine availability to OXPHOS regulation [46]. Further development of novel technologies, such as submicron single-cell mass spectrometry, will be required to resolve nucleotide pools spatially and to better define the nucleotide flux between cellular compartments [17,47].

### Mitochondrial nucleotide transport

Analogous to the nuclear membrane, nucleotides cross the OMM through aqueous pores formed by abundant voltage-dependent anion channels (VDACs). The outer membrane is, therefore, porous to most small molecules. VDAC can limit adenine nucleotide transport in isolated mitochondria, particularly if in a low conductance state which limits the transport of anions across the OMM [54]. A number of post-translational mechanisms, including interactions with cytosolic and mitochondrial proteins, have been proposed to alter VDAC permeability [55], and VDAC3 tethers the pyrimidinosome to mitochondria [25]. However, the extent to which VDAC pores dynamically regulate mitochondrial ADP/ATP flux or access to other nucleotides remains unclear.

Unlike the porous OMM, nucleotide transport across the impermeable IMM requires dedicated transport proteins, which are fundamental regulators of compartmentalised nucleotide metabolism. dNs and (deoxy)nucleotides in all three phosphorylation states ((d)NMPs, (d)NDPs and (d)NTPs) can apparently enter mitochondria. It remains unclear whether there is preferential uptake of certain phosphorylated species. Most mitochondrial metabolite transporters belong to the solute carrier 25 (SLC25) family of small metabolite transport proteins, which share a common structure and alternate access transport mechanism to exchange metabolites across the IMM [56]. Approximately one quarter of the 53 SLC25 family proteins remain to be characterised; however, multiple mitochondrial nucleotide carriers have been identified (Figure 2).

#### Purine nucleotide transport

Mammalian mitochondria contain four SLC25 carriers that import and export equimolar amounts of ADP and ATP, respectively. These abundant carriers, therefore, exchange the substrate and product of ATP synthase. Their relative expression is tissue-dependent, and structural studies of SLC25A4 (ADP/ATP Translocase 1) have informed most of our understanding of SLC25 carrier transport mechanism. Dominant and recessive mutations of SLC25A4 are associated with diverse pathologies linked with mtDNA mutation and depletion [57]. The ADP/ATP carriers have the capacity to transport deoxynucleotides dADP and dATP, which possibly explains why mtDNA is depleted upon SLC25A4 mutation [58].

Four paralogous ATP-Mg/Pi carriers drive mitochondrial ATP import in exchange for phosphate [59,60] and, unlike the ADP/ATP carriers, they can alter the mitochondrial adenine nucleotide pool in response to metabolic demands [61]. Known collectively as short calcium-binding mitochondrial carriers [62], the transport activity of three mitochondrial ATP-Mg/Pi carriers requires calcium binding to four EF hands exposed to the IMS (Figure 2). The control of the mitochondrial adenine pool by the ATP-Mg/Pi carriers likely influences a number of metabolic pathways by regulating adenine nucleotide-dependent enzymes in the matrix [63] and by influencing Ca^2+^ homeostasis [64,65].

Mutant ATP-Mg/Pi carriers have not been associated with mtDNA depletion; however, pathogenic mutations in *SLC25A24* lead to a connective tissue disease called Fontaine progeroid syndrome [66,67]. Further work is required to understand the impacts of pathogenic mutations on SLC25A24 transport activity and how this severe developmental disorder is linked to disturbed mitochondrial adenine nucleotide transport.

In comparison with adenine nucleotides, far less is understood about the regulation of mitochondrial guanine nucleotide transport. A dedicated mitochondrial GTP/GDP carrier has been characterised in yeast [68], but the only SLC25 carriers known so far to transport guanine nucleotides in mammals are two mitochondrial carriers, SLC25A33 and SLC25A36, which have, otherwise, been characterised as pyrimidine nucleotide carriers [69,70].

#### Pyrimidine nucleotide transport

SLC25A33 and SLC25A36 are closely related to the yeast mitochondrial pyrimidine carrier Rim2, which is essential for mtDNA maintenance and non-fermentative growth [71,72]. SLC25A33, SLC25A36 and Rim2 share similar substrate specificity for pyrimidine and guanine (deoxy)nucleotides [69,70,72]. Accordingly, expression of either mammalian pyrimidine carrier is sufficient to restore mtDNA and mitochondrial respiration in Rim2-deficient yeast [69]. However, loss of both SLC25A33 and SLC25A36 only causes mild mtDNA depletion in proliferating cell lines due to sufficient mitochondrial deoxynucleotide salvage in cultured cells [73]. Increased expression and stability of SLC25A33 at the IMM is sufficient to up-regulate mtDNA synthesis in cultured cells, likely due to increased mitochondrial uptake of pyrimidine dNTPs [44,74]. It remains unclear to what extent the dynamic regulation of pyrimidine/guanine nucleotide import into mitochondria controls mitochondrial gene expression across different cell types and tissues. Mutations in *SLC25A36* were identified in two patients with hyperinsulinism/hyperammonaemia (HI/HA) syndrome [75,76], but mtDNA levels appeared to be unperturbed in affected tissue [76]. Instead, dysfunctional SLC25A36 in individuals with HI/HA syndrome was linked to reduced mitochondrial GTP levels, which may lead to hyperactivated glutamate dehydrogenase [75].

It is likely that the road map for mitochondrial nucleotides across the mitochondrial inner membrane remains incomplete and novel nucleotide transporters await discovery. Transporter redundancy is a common feature across mammalian solute carriers, and nucleotide carriers are no exception. It is important to consider that other mitochondrial transporters exchange nucleotides with other metabolites, underlining the promiscuity of some solute carriers [77]. For instance, SLC25A42 imports coenzyme A in exchange for (deoxy)adenine nucleotides [78], which highlights further the complexity of mitochondrial adenine nucleotide homeostasis. Metabolite transporters outside the SLC25 family have also been implicated in mitochondrial nucleotide transport. Mutations in the enigmatic IMM protein, MPV17, cause mtDNA depletion syndromes (MDS) [79] associated with the depletion of dNTPs [80]. MPV17 has been suggested to facilitate mitochondrial uptake of dTMP [81], but evidence of transporter activity is lacking and other studies suggest that MPV17 oligomers may harbour channel-like activity [82–84].

### Mitochondrial nucleotide salvage

Mitochondria cannot always import sufficient dNTPs or NTPs from the cytosol and therefore have the capacity to phosphorylate precursor nucleotides in the mitochondrial nucleotide salvage pathway. Many of the mitochondrial nucleotide transporters introduced above can import monophosphate ((d)NMPs) and diphosphate ((d)NDPs) nucleotides into the mitochondrial matrix prior to further phosphorylation by nucleotide kinases. Mitochondrial dNTP supply appears to depend predominantly on mitochondrial nucleotide salvage in differentiated, postmitotic tissue where RNR activity and cytosolic dNTP production are suppressed [51]. Whether mitochondria also depend on NTP salvage to a greater extent in these tissues is not understood. 

The protein structures and enzymatic activity of mitochondrial salvage pathway enzymes are largely shared with conserved enzymes in the cytosolic nucleotide salvage pathway. Salvage pathway enzymes have been intensely studied largely due to their roles in the metabolism and activation of nucleoside analogue drugs used in antiviral and antitumour therapies. These kinases catalyse the transfer of γ-phosphate from donor NTPs (predominantly ATP) to the ribose group of acceptor dNs at the 5′-OH or onto pre-existing α or β phosphate groups of acceptor (d)NMPs and (d)NDPs, respectively [85]. Intriguing differences exist between the regulation of dNTP and NTP salvage within mitochondria, and we are only beginning to understand the cell states and metabolic contexts that influence mitochondrial dependency on nucleotide salvage.

#### Salvage step 1: deoxynucleoside to dNMP

Most attention has been placed on the mitochondrial salvage of dNTPs, specifically because disturbed mitochondrial dNTP salvage can cause MDS. Since nucleotide salvage from free deoxynucleobases has not been described in mitochondria, the first step of mitochondrial deoxynucleotide salvage is the phosphorylation of dNs to (deoxy)nucleoside monophosphates (dNMPs). Interestingly, there is currently no evidence of ribonucleoside phosphorylation to NMP in mitochondria, whereas UMP and cytidine monophosphate (CMP) can be synthesised from uridine and cytidine in the cytosol by uridine–cytidine kinase 1/2 [2]. dNs can be imported into mitochondria by the SLC29 family members of equilibrative nucleoside transporters (ENT) 1 and 3. ENT1 and 3 have been localised to both the plasma membrane and mitochondrial membrane where their expression level associates with mitochondrial toxicity of nucleoside analogues used in antiviral and anticancer therapy [86]. Tagged human ENT1, but curiously not rodent ENT1, localises to mitochondria and ENT1 expression facilitated mitochondrial uptake of uridine and the nucleoside analogue fialuridine, which causes mitochondrial toxicity [87,88]. ENT3 was also observed at mitochondria and is linked to the mitochondrial uptake of a panel of nucleoside analogues, including 2′-,3′-dideoxycytidine (ddC) which inhibits POLγ leading to mtDNA depletion and mitochondrial toxicity in patients [89,90]. The expression of ENTs can be up-regulated to support increased dependence on nucleotide salvage. For example, inhibition of OXPHOS and purine synthesis enhances ENT1 expression [28]. It remains unclear whether the localisation of ENT1/3 to mitochondria is dynamically regulated in conditions that limit cytosolic *de novo* nucleotide synthesis. Understanding this may help counteract mitochondrial toxicity of nucleoside analogues.

dNs are phosphorylated to dNMPs in the mitochondrial matrix by thymidine kinase 2 (TK2) and deoxyguanosine kinase (DGUOK). TK2 irreversibly phosphorylates pyrimidine dNs (deoxyuridine (dU), deoxycytidine (dC) and thymidine (dT)), while DGUOK preferentially phosphorylates purine dNs deoxyadenosine and deoxyguanosine. TK2-mediated synthesis of dUMP and dTMP can be opposed by dephosphorylation catalysed by the mitochondrial deoxyribonucleotidase, dNT2 [91]. This activity may be important to limit dTTP accumulation in mitochondria and to avoid incorporation of dUTP into mtDNA [92].

Mutations in *TK2* or *DGUOK* cause MDS with clinically distinct symptoms associated with mtDNA depletion [53]. *TK2* mutations cause skeletal myopathy associated with respiratory chain defects in the skeletal muscle [93,94]. It remains unclear why skeletal muscle is severely affected by the inhibition of TK2, while other post-mitotic and high-energy demanding tissues appear normal in patients. One possibility is that normal skeletal muscle has a low basal activity of TK2 but a high demand for mtDNA-encoded subunits of the respiratory chain [95] or compensatory up-regulation of mitochondrial transcription in some tissues as reported in *Tk2* mutant mice [96]. Mice harbouring a disease relevant *Tk2* mutation have impaired mtDNA maintenance and respiration in the brain and spinal cord, which can be ameliorated by treatment with pyrimidine nucleotides [97,98]. The majority of patients with mutations in *DGUOK* suffer from early onset neurological dysfunction and liver failure [99,100]. Several tissues with dysfunctional DGUOK activity have depleted mtDNA, which was reported to be most severe in the livers of Dguok-deficient mice [101]. Purine nucleotide supplementation could restore mtDNA levels in *DGUOK* mutation patient-derived cells [102,103] and Dguok knockout zebrafish [104], but further work is required to explore this therapy in the clinic. 

#### Salvage step 2: (d)NMP to (d)NDP

Mitochondria appear to contain several kinases that phosphorylate both dNMPs and NMPs to dNDPs and NDPs collectively known as nucleoside monophosphate kinases (NMPKs). Three isoforms of adenylate kinase (AK) localise to mitochondria and are expected to catalyse ADP synthesis from AMP using ATP or GTP as phosphate donors [105]. AK3 and AK4 are imported to the mitochondrial matrix [106], while AK2 is in the IMS [107]. A guanine nucleotide-specific NMPK has not been described in mitochondria. However, the use of GTP as a phosphate donor by the promiscuous AK, AK4, would produce GDP, as well as ADP (i.e. AMP + GTP = ADP + GDP) [106,108]. Phosphorylation of (d)CMP and UMP is catalysed by the mitochondrial matrix protein CMPK2 [109], and phosphorylation of dTMP to dTDP is likely catalysed by mitochondrial localised deoxythymidylate kinase (DTYMK) [110,111]. Curiously, CMPK2 appears to preferentially phosphorylate dUMP, whereas supraphysiological Km values in the mM range for the phosphorylation of UMP and CMP have been reported [109]. Furthermore, a kinase activity assay with recombinant human CMPK2 implicated that it may act in the next step of nucleotide salvage and preferentially phosphorylate CDP and UDP [112]. As discussed later in this review, CMPK2 is an interferon-stimulated gene that remains an enigmatic enzyme with apparent kinase-independent roles. Mammalian DTYMK appears to be dual localised between the mitochondria and cytosol, unlike mitochondrial TK2 which is a distinct isomer to cytosolic TK (see step 1 above). However, the precise sub-mitochondrial localisation of DTYMK and contribution of intra-mitochondrial dTMP phosphorylation to mitochondrial dTDP pools remains to be determined [113].

#### Salvage step 3: (d)NDP to (d)NTP

Nucleoside diphosphate kinases (NDPKs) catalyse the final step of nucleotide salvage. These enzymes provide a high-energy phosphohistidine intermediate to transfer the terminal phosphate from a donor nucleotide (usually ATP) onto (d)NDP to generate (d)NTP [114]. Ten cellular NDPKs belong to the diverse NME (non-metastatic) protein family of which the canonical members have promiscuous NDPK activity and can phosphorylate all (d)NDPs as substrates. Three members, NME3, NME4 and NME6, localise to mitochondria [115].

NME4 and NME6 are imported into mitochondria, while NME3 is tethered to the OMM via the phospholipid phosphatidic acid. NME3 regulates mitochondrial dynamics via protein–protein interactions that do not depend on its NDPK activity [116–118]. There is no evidence currently to suggest that NME3 plays a direct role in supplying mitochondrial (d)NTPs. NME4 has been identified in the matrix and IMS and reportedly binds to the phospholipid cardiolipin in the IMM [119–121]. In the matrix, NME4 may contribute to dGTP production via interaction with the succinyl-CoA synthetase [122,123], while in the IMS, NME4 can supply GTP to the dynamin-like GTPase OPA1 to regulate mitochondrial fusion [124]. NME4 is expected to function as a canonical hexameric NDPK and to synthesise other (d)NTPs beside GTP, but this has not been demonstrated experimentally. It was proposed that the mtDNA depletion caused by deleterious mutation of succinyl Co-A synthase gene *SUCLA2* might also be related to dNTP supply by account of its interaction with NME4 [125].

NME6 is found exclusively within the mitochondrial matrix and is regarded as a non-canonical NDPK family member because it does not oligomerise into hexameric complexes. Instead, NME6 phospho-transfer activity depends on the ATPase activity of its interaction partner, RCC1L [126,127]. Loss of NME6 results in the decline of mitochondrial pyrimidines, predominantly dCTP and CTP [73], which is consistent with *in vitro* NDPK activity assays that found NME6 to phosphorylate UDP and CDP [127,128]. Cultured cells lacking NME6 can sustain mtDNA synthesis, likely via SLC25A33 and SLC25A36-mediated dNTP import [73]. However, loss of NME6 limits the accumulation of mitochondrial transcripts and inhibits mitochondrial translation, which can be rescued by pyrimidine nucleotide supplementation. NME6 therefore appears to be particularly important for the supply of pyrimidine NTPs destined for mtRNA synthesis in some cell lines. It remains to be seen whether NME6 is indeed a pyrimidine-specific NDPK or whether alternative sources of ATP, GTP and dTTP can bypass NME6.

## Are mitochondrial DNA and RNA local nucleotide reservoirs?

It is intriguing to speculate to what extent mitochondrial nucleic acid turnover contributes to the intrinsic nucleotide pool. The turnover rates of mtDNA vary across tissue. For example, mtDNA half-life was measured at almost 7 days in rat liver compared with over 30 days in rat brain [129]. Turnover likely results from a combination of whole-sale mitochondrial digestion in the lysosome and mitochondrial nucleases, which can process mtDNA fragments into deoxynucleotides that may be reused for new mtDNA synthesis. However, unless a high proportion of mtDNA molecules are degraded within mitochondria at a given time, it is unlikely that mtDNA hydrolysis contributes to a significant proportion of the mitochondrial deoxynucleotide pool. By comparison, mitochondrial mtRNA is far more abundant and is degraded much faster with a median half-life for mitochondrial mRNA calculated around 75 min [130]. In bacteria, nuclease-dependent hydrolysis of mRNA, and to a lesser extent rRNA, releases NMPs which are processed and further phosphorylated for reuse [131]. Mitochondria contain several nucleases that combine to degrade RNA to individual NMPs. The mitochondrial degradosome, which includes the RNA helicase SUV3 and the polynucleotide phosphorylase, PNPase, cleaves mitochondrial RNA species above 4 nucleotides in length including antisense RNA, double-stranded RNA and mRNA into short oligonucleotides [132–134], which can be processed by other mitochondrial nucleases such as ExoG into dinucleotides [135,136]. The mitochondrial ribonuclease REXO2 cleaves DNA and RNA dinucleotides [137], which likely represents the terminal stage of mtDNA and mtRNA degradation within mitochondria [138]. REXO2 activity, therefore, releases NMPs, which can be used for mitochondrial transcription [138]. The dinucleotide substrates of REXO2 can stimulate mitochondrial transcription initiation by the mtRNA polymerase POLRMT [138], which may confound future efforts to quantify the contribution of REXO2-dependent NMP recycling towards mitochondrial transcription.

## Cytosolic nucleotide metabolism and mitochondrial disease

The coupling of cytosolic and mitochondrial nucleotide pools (Box 1) suggests that altered dNTP metabolism in the cytosol can have direct consequences for mitochondrial dNTP homeostasis and mitochondrial genome maintenance. Indeed, disturbed nucleotide metabolism in the cytosol can drive pathogenic imbalances between mitochondrial nucleotides. Inactivating mutations in the gene encoding thymidine phosphorylase, *TYMP*, causes mitochondrial neurogastrointestinal encephalomyopathy (MNGIE), which is characterised by mtDNA mutation and depletion in patients [139,140]. Thymidine phosphorylase is required for the phosphorolysis of dT and dU to their respective bases and its inhibition results in elevated plasma levels of dT and dU [141,142]. In-organelle assays suggest that ‘thymidine overload’ and elevated mitochondrial dTTP result in a concomitant depletion of mitochondrial dCTP, which ultimately impairs mtDNA replication [143].

*RRM2B* encodes the cell-cycle independent and DNA-damage inducible subunit of the heterodimeric RNR complex also known as p53R2. *RRM2B* mutation causes MDS associated with the depletion of dNTPs, specifically dATP and, to a lesser extent, dGTP, in postmitotic tissue [144,145]. A diverse spectrum of symptoms is reported in patients with *RRM2B* mutations, including progressive external ophthalmoplegia, myopathy and MNGIE [146–148]. Mutation of *RRM1*, which encodes the larger catalytic subunit of RNR, was also identified in individuals with mtDNA depletion, myopathy, ptosis and ophthalmoplegia [149]. Together, the clinical association between impaired RNR activity and mtDNA depletion argues that in certain tissues, the mitochondrial dNTP salvage pathway is unable to compensate for cytosolic loss of dNTP supply, a notion which is supported by computational modelling of mitochondrial nucleotide metabolism enzyme kinetics [150]. Some cells and single-cell organisms may also depend on the recycling of cytosolic dNTPs by autophagy to maintain a sufficient dNTP supply for mtDNA replication [151].

The rate of cellular dNTP catabolism has a strong impact on mitochondrial dNTP supply and may contribute to the tissue specificity of mitochondrial disease. For instance, the deoxynucleotide triphosphohydrolase SAMHD1 limits the accumulation of dNTPs in slow-dividing cells, and SAMHD1-dependent conversion of dNTPs to dNs can exacerbate the depletion of dGTP in *DGUOK* mutant cells [152,153]. Indeed, SAMHD1 activity is up-regulated in post-mitotic tissue, which causes the selective depletion of dATP and dGTP observed in RRM2B-inhibited cells [145]. Inactivation of SAMHD1 leads to increased cellular dNTP levels which suppresses the erroneous incorporation of ribonucleotides into mtDNA [154] and is associated with increased mtDNA copy number [155]. Importantly, mutation of *SAMHD1* is a cause of Aicardi–Goutières syndrome, an early-onset inflammatory disorder [156]. However, the mechanistic links between SAMHD1 and innate immune regulation lack clarity and may relate to SAMHD1 regulation of dNTP homeostasis or its RNA exonuclease activity [157].

It is also important to consider the impact of nucleoside analogues on mitochondrial gene expression. Nucleoside analogues are a class of anti-metabolites used in antiviral and cancer therapy, which usually require intracellular phosphorylation by salvage pathway enzymes prior to incorporation into cellular and viral DNA. Overexpression of mitochondrial nucleotide salvage pathway enzymes can activate nucleoside analogues within mitochondria and support their cytotoxicity by inhibiting mitochondrial function [158]. The mitochondrial uptake of cytosolic-activated nucleoside analogues can also inhibit mtDNA synthesis. This was found to cause severe mitochondrial toxicity in individuals treated with antiviral compounds such as ddC [159]. Once phosphorylated by cytosolic dC kinase, ddC enters mitochondria and inhibits the mtDNA polymerase POLγ, resulting in mtDNA depletion and clinical phenotypes commonly associated with mitochondrial disease [160–163]. While mitochondrial toxicity renders ddC intolerable for antiviral therapy, ddC has been proposed as a cytotoxic drug in acute myeloid leukemic cells that express high levels of dC kinase [164]. Importantly, recent results show in the case of the pyrimidine nucleoside analogue, 5-fluorouracil, nucleoside analogue therapies can have differential effects on mitochondrial function and metabolism that do not stem from inhibited mtDNA replication [44,165].

## Mitochondrial nucleotide balance and cell signalling

This review has largely focussed on how the supply and metabolism of nucleotides must be maintained to support the expression of the mitochondrial genome and ultimately mitochondrial respiratory capacity. Mitochondria have also emerged as core organelles in nucleic acid sensing and the control of innate immune signalling [166]. Recent work highlights that mitochondrial nucleotide supply is linked to inflammation and innate immune pathways. When released from the mitochondrial matrix, mtDNA and mtRNA are recognised as damage associated molecular patterns by cytosolic and endosomal receptors, which triggers a myriad of downstream inflammatory responses, including the expression of interferons and interferon stimulated genes (ISGs). Various genetic and environmental stressors have been shown to trigger the release of mitochondrial nucleic acid via different mechanisms, which are reviewed elsewhere [167,168]. A link between mitochondrial nucleotide flux and mtDNA-dependent inflammation was first suggested in stimulated macrophages. Macrophages primed with lipopolysaccharide (LPS) up-regulate the mitochondrial nucleotide kinase, CMPK2, which is itself a type-I ISG induced upon infection [169]. Enhanced CMPK2 catalytic activity boosts mtDNA synthesis in stimulated macrophages and triggers the cytosolic exposure of oxidised mtDNA which activates the NLRP3 inflammasome complex. Other infections, such as dengue virus, drive mtDNA oxidation and release without increasing mtDNA synthesis [170]. However, it remains unclear whether enhanced CMPK2 expression stimulates mtDNA replication by driving flux through mitochondrial pyrimidine deoxynucleotide salvage pathway, although LPS-induced mtDNA synthesis also depended on the presence of NME4. Interestingly, the mitochondrial NDPKs, NME4 and NME6, were both identified as positive regulators of mtDNA-dependent inflammasome activation [171]. NME4 regulates lipid metabolism [172,173], which conceivably could regulate oxidative stress and mitochondrial integrity independent of mtDNA synthesis.

Enhanced pyrimidine nucleotide uptake into mitochondria by increased expression of the pyrimidine nucleotide carrier, SLC25A33, also triggers an mtDNA-dependent innate immune response [44]. Excessive uptake of pyrimidine nucleotides into mitochondria somehow triggers the release of mtDNA into the cytosol, which stimulates ISG expression downstream of the dsDNA receptor cGAS and STING. Inhibition of pyrimidine synthesis in the cytosol is also sufficient to trigger an analogous mtDNA-dependent immune response, which highlights the link between cellular nucleotide homeostasis and maintenance of the mitochondrial genome. Furthermore, nucleotide imbalance, specifically of dNTPs between mitochondria and cytosol, can lead to nuclear DNA damage and replication stress. In mouse-derived pluripotent stem cells, dysregulated mtDNA replication and excessive dNTP uptake by mitochondria lead to a concomitant depletion of dNTP pools required for nuclear genome replication and repair [45]. The coupling between mtDNA replication defects and cellular dNTP homeostasis is likely to depend on cell-type and developmental stage specific contexts [174].

## Conclusions and future perspectives

Mitochondria support the production of nucleotides required for cell growth and survival, and they depend on nucleotides for the replication and expression of the mitochondrial genome. It is important to understand how mitochondria import and metabolise deoxynucleotides because disturbed mitochondrial nucleotide supply can lead to severe diseases caused by mtDNA depletion. The restoration of dNTP levels using nucleoside bypass therapy continues to be explored as a treatment option for patients with MDS [175]. However, it remains a challenge to understand how the supply and balance between individual nucleotide species is differentially regulated across different tissues. Advancement in spatial metabolite measurements and metabolic tracing *in vivo* may help us to understand how mitochondrial nucleotide supply is maintained across cell types and why mutation of nucleotide metabolism enzymes leads to diverse symptoms in patients.

Can mitochondria sense changes in nucleotide availability? Cellular nucleotide imbalance caused by excessive accumulation of individual dNTPs induces nuclear DNA replication stress. This halts cell division in S phase and signals cells to boost dNTP availability in order to permit cell cycle progression [176], potentially via up-regulation of RNR activity [177]. It remains unclear whether nuclear replication stress signalling affects mitochondrial nucleotide uptake and salvage besides up-regulating RNR activity or whether other mechanisms sense nucleotide levels locally within mitochondria. For instance, a decline in mitochondrial dNTP levels can cause mitochondria-to-nucleus retrograde signalling indirectly via mtDNA depletion [178].

Finally, it is important to consider whether mitochondrial nucleotide supply is regulated dynamically in response to different environmental conditions and metabolic stress. As highlighted in this review, mitochondria can influence cytosolic nucleotide synthesis pathways, which could protect their own nucleotide supply routes. Mitochondrial nucleotide balance may also be modulated directly via the regulation of nucleotide transport. For example, expression of the mitochondrial pyrimidine nucleotide carrier, SLC25A33, appears to be up-regulated by mTORC1 [70] and fine-tuned post-translationally at the IMM by proteolysis [44]. Further work will reveal how mitochondrial solute carriers and nucleotide salvage pathways are integrated in the signalling programmes that control nucleotide metabolism. This may provide new pathways to target not only in mitochondrial disease but also in pathological scenarios associated with altered nucleotide metabolism, including cancer and autoimmune disease.

## Data Availability

There are no data associated with this article.

## Abbreviations

AK: adenylate kinase
CMP: cytidine monophosphate
DGUOK: deoxyguanosine kinase
DHODH: dihydroorotate dehydrogenase
DTYMK: deoxythymidylate kinase
ENT: equilibrative nucleoside transporter
HI/HA: hyperinsulinism/hyperammonaemia
IMM: inner mitochondrial membrane
IMS: intermembrane space
ISGs: interferon stimulated genes
LPS: lipopolysaccharide
MDS: mtDNA depletion syndromes
MNGIE: mitochondrial neurogastrointestinal encephalomyopathy
NDPKs: nucleoside diphosphate kinases
NMPKs: nucleoside monophosphate kinases
OMM: outer mitochondrial membrane
RNR: ribonucleotide reductase
SLC25: solute carrier 25
TK2: thymidine kinase 2
UMP: uridine monophosphate
VDAC: voltage dependent anion channel
dC: deoxycytidine
dNMP: (deoxy)nucleoside monophosphate
dNs: deoxynucleosides
dT: thymidine
dU: deoxyuridine
dUMP: thymidine monophosphate
dUMP: deoxyuridine monophosphate
ddC: dideoxycytidine
mtDNA: mitochondrial DNA
mtRNA: mitochondrial RNA
